# Benign tumours leading to total penile denudation treated with Manuka honey dressings: A case report and review of literature

**DOI:** 10.1016/j.ijscr.2019.07.009

**Published:** 2019-07-19

**Authors:** Amalie Sylvester-Hvid, Magnus B. Avnstorp, Tobias Fjeld, Bjørn Crewe

**Affiliations:** Department of Plastic- and Breast Surgery, University Hospital Zealand, Sygehusvej 10, DK-4000, Roskilde, Denmark

**Keywords:** Penile denudation, Penile reconstruction, Manuka honey, Wound infection, Split-thickness skin graft, Balanitis

## Abstract

**Introduction:**

Penile denudation is a devastating condition often reconstructed with a split-thickness skin graft(STSG). As this kind of reconstruction is challenging, we present an non-invasive treatment using Manuka honey dressings with a satisfying result. This was performed as a salvage procedure after failed STSG.

**Case:**

A 55-year-old non-smoking male was admitted from his general practitioner with a newly onset of phimosis and lower urinary tract symptoms. Benign tumours complicated with infection were found on all segments of the penis causing dehiscence of the skin. After surgical removal of tumours and an unsuccessful STSG, Manuka honey dressings was used. Full sexual function was regained, and the patient was satisfied with the result.

**Discussion:**

Alternatives to STSG are full-thickness skin graft using the inguinal or scrotal borrowing method, or using a dermal matrix before a STSG.

A rediscovered method is using Manuka honey, with its unique combination of bactericidal, anti-inflammatory and healing-promoting properties. A wide range of wound types may benefit from Manuka honey dressings.

A recent Danish in vitro study on honey derived from various Danish floras even shows high antibacterial effect superior to commercial medical grade honey. Considering a growing resistance to antibiotics, medical honey may contribute as a alternative to extensive wound care.

**Conclusion:**

We successfully treated a penile denudation with Manuka honey following a failed STSG. Wounds, ulcers, and burns may be infected, and can be challenging, time consuming, and expensive to treat. Manuka honey may be a good alternative to reconstructive surgery and can be managed on an out-patient basis.

## Highlights

• Penile denudation can be treated either with reconstructive surgery or conservatively using Manuka honey dressings.
• Split-, full-thickness skin graft or local skin flap are surgical reconstructive possibilities regarding penile denudation.
• Methylglyoxal is responsible for the antibacterial activity in Manuka honey contrarily to other types of honey.
• Manuka honey treatment requires a minimum of medical training and be used in the out-patient clinic.
• A wide variety of wound types can benefit from Manuka honey dressings.

## 1 Introduction

Penile denudation is a rare and devastating injury with potential loss of sexual function and imperilled aesthetic appearance. Not many cases have been reported, although the aetiologies revolve around animal bites, trauma from the use of power-driven tools, burns, infections, and circumcisions.

A split-thickness skin graft (STSG) is the conventional way of treating patients with penile denudation. When a STSG in this difficult anatomic region is unsuccessful, alternative conservative treatment must be considered.

Over the last couple of decades, a scientific explanation of honey’s effectiveness has been developed. A pH of around 3.2–4.5 promotes healing by increasing the release of oxygen from haemoglobin as well as decreasing protease activity. The high osmolarity due to the sugar content in honey creates an outflow of lymph. In addition the antibacterial properties of methylglyoxal makes Manuka honey wound dressings a good alternative to other wound care products [1].

We present a case of total penile denudation due to several benign tumours with deep infected ulcers treated with Manuka honey. Because of the simple use, effectiveness, low cost, and wide applicability, we find it relevant to present this treatment as a non-invasive treatment option for challenging wounds in the genital region as it is not well-described elsewhere. To the authors’ knowledge this is the first reported case of penile denudation treated conservatively with Manuka honey after failed attempt with split skin graft. This case report has been reported in line with the SCARE criteria [2]

## 2 Presentation of case

A 55-year-old non-smoking male with known hypertension, gout, and Non-Insulin-Dependent Diabetes Mellitus, NIDDM, was admitted from his general practitioner with phimosis, lower urinary tract symptoms, and a spontaneous infectious fissure in the preputium. First thought to be a severe case of balanoposthitis, the patient was circumcised. Intraoperatively the distal 2/3 of the penile shaft had undermining of the skin due to three bleeding, pus-filled ulcers. Tumours were found at the root of the penis, on the shaft, and on the glans. The tumours were removed and treatment with cefuroxime was initiated in combination with metronidazole in accordance with cultures showing growth of anaerobic bacteria and haemolytic streptococci group B and G. Histological examination of the removed tumours showed pseudocarcinomatous epithelial hyperplasia, but no malignancy.

**Fig. 1:**
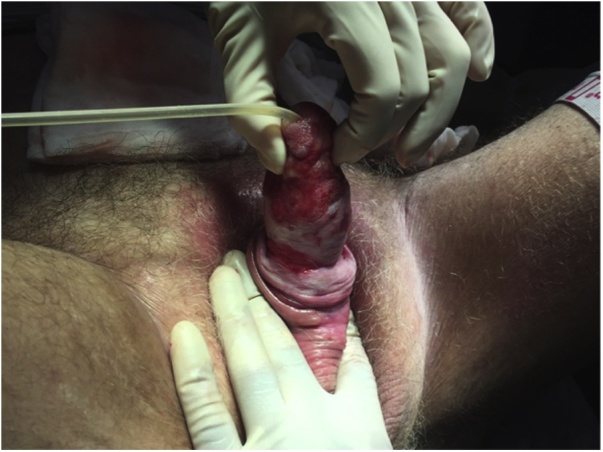
After failed split-thickness skin graft and before second round of Manuka honey dressings.

**Fig. 2:**
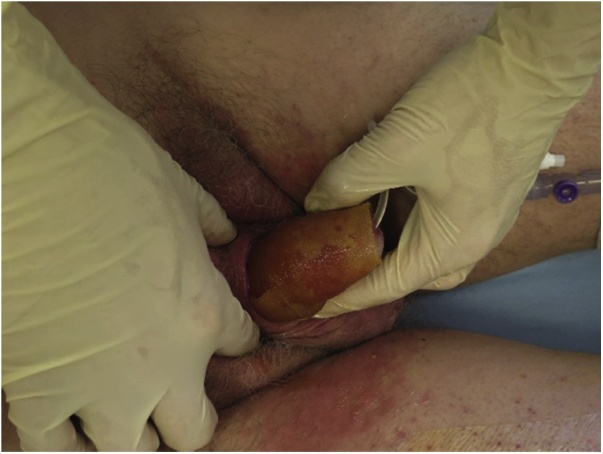
The Manuka honey dressing before aquacel and regular gauze are added.

**Fig. 3:**
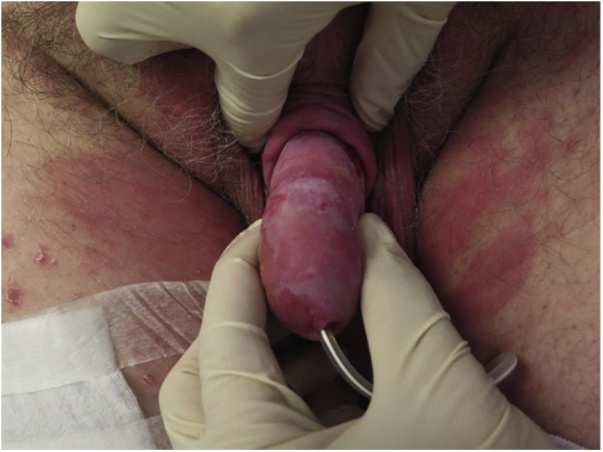
Two month with continued honey treatment following failed skin graft.

The patient was referred to our department of Plastic Surgery. We initiated treatment with Manuka honey. After 2 weeks of conservative wound treatment with Manuka honey dressings and antibiotics, granulation tissue was achieved. A STSG from the thigh was performed in general anaesthesia. Unfortunately the graft was unsuccessful and one week after the procedure the graft was removed due to missing adherence of skin graft (Fig. 1). Conservative treatment with only Manuka honey dressings was carried on (Fig. 2). The patient changed the dressings himself every other day without complications, recurrent infections or associated pain, and a steady healing process was observed. A Foley catheter was used throughout the whole treatment period to ensure control and avoid wetting of the dressings. Fifty-two days after we initiated treatment at our department, the penis was completely healed (Fig. 3).

The patient regained full sexual function and was satisfied with both the functional as well as the aesthetic result.

## 3 Discussion

Several surgical methods exist in the treatment of penile denudation - the most popular and simple being the split-thickness skin graft [3]. Other valuable options are use of dermal matrix, full-thickness skin graft (FTSG) or a local flap.

Due to the physiological tendency of the corpora cavernosa to change size during both day and night time, the challenge with a STSG is to keep the graft aligned and proximate consistently. This can be solved either by an elastic compressive “chimney dressing” keeping the penis in erect position [3], or by placing stay-sutures from the base of glans to the inguinal region [4] to hold the penis and thereby the graft in place.

We found a successful healing using Manuka honey (Activon®) dressings applied directly onto the denuded penis in a chimney dressing. It contained three layers of Manuka honey impregnated alginate fibre-dressings (Algivon Plus®) with aquacel, regular gauze, and mesh briefs.

We achieved an unsuccessful result using a STSG, held in place using the chimney dressing method without any stay-sutures.

Triana JP has described a two-step method using dermal matrix later followed by a STSG. The dermal matrix is attached with staples and kept in place for 4–5 weeks, followed by a STSG. The study exhibits same cosmetic and functional results as a STSG without dermal matrix or a FTSG [5].

A full thickness skin graft can be done with “the inguinal borrowing method”. Two incisions and a tunnel along the iliac fossa are dissected. The penile corpus is covered while the glans protrudes through the lateral incision. In a stepwise manner the penis is dissected and covered the following weeks [6].

**Fig. 4:**
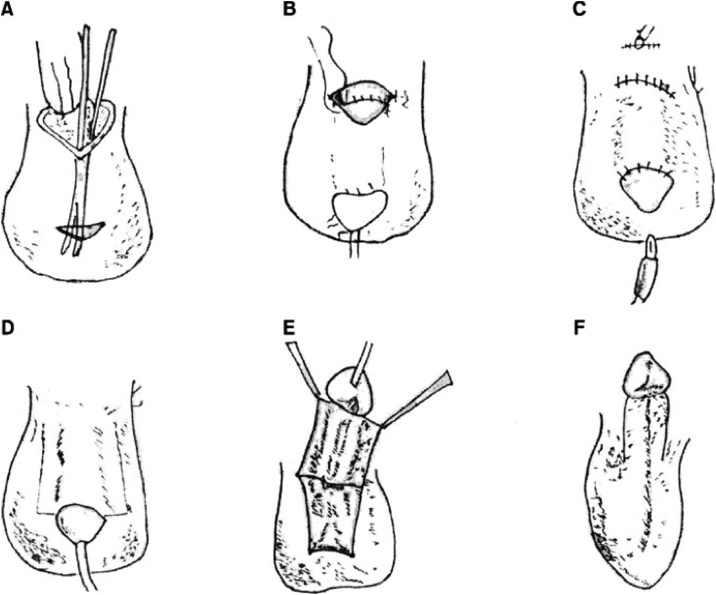
The burrowing method. A: Two incisions and a tunnel in the sub-cutis of the scrotum are dissected. B: The denuded penis is placed in the pocket, leaving the glans on the outer skin. C: The incision is closed and the catheter removed. D: After 6–8 weeks a catheter is reinserted to facilitate manipulation of the penis. Full thickness incisions over the scrotal skin along the penile shaft are performed. E: The penile shaft lifted off the scrotal sac along with the full thickness skin covering it. F: The skin edges are sutures separating the penis from the scrotal sac. Donorsite is sutured.

Last valuable option is a local scrotal flap – “the scrotal borrowing method” [7]. On the anterior scrotal surface a flap is raised to match skin loss in both vertical and circumferential direction. A buttonhole incision is made at the base of the flap and the penis is brought through the hole, so the flap covers the dorsal side. Sutures are placed to match the raphe and frenulum (Fig. 4). Disadvantages of this flap are risk of flap necrosis, infections, stricture of the urethra, fistulas and that the fixation of that penis for 6–8 weeks can cause some discomfort for the patient [7].

An antibacterial Manuka honey dressing can be a valuable supplement in the pre- and post-operative care, and it can advantageously be used prior to fixating the STSG [8].

We found it reasonable to continue the conservative treatment with Manuka honey following the failed reconstruction with a STSG, as the healing progress promoted by honey had been uncomplicated so far. The patient was satisfied with this choice of treatment and the result. Otherwise the other described surgical procedures could have been useful reconstructive options.

Manuka honey has previously been found effective in the treatment of extensive dehiscent, and infected abdominal defects [9]. Good results have also been achieved in the treatment of, among others, Fournier’s gangrene, decubitus, diabetic leg ulcers, and burns [10,11].

A clinical study on honey and silver sulfadiazine dressings in the treatment of second degree burns found an early histological subsidence of inflammatory changes, superior infection control and fast wound healing, when honey was used [12].

More than 300 varieties of honey exist. Most honeys exhibit their antibacterial activity through hydrogen peroxide generated by glucose oxidase, which is activated by wound exudate diluting the honey. Unfortunately with the wound exudate comes catalase, which decomposes the hydrogen peroxide [1]. In 2008, methylglyoxal was identified as being responsible for the superior antibacterial activity of New Zeeland Manuka honey, in part because of the resistance to catalase [13].

It should be noted that medical grade honey is sterilized with gamma radiation to eliminate bacterial spores without diminishing the antibacterial effect of the honey [14].

A new Danish study [15] on honey derived from different Danish flora tested in vitro showed a high antibacterial effect on *Staphylococcus aureus*, *Staphylococcus epidermidis* and *E. coli*. Some of the Danish honeys had an effect on the pathogens, which were similar or even superior to commercial medical grade honey, primarily due to its high content of hydrogen peroxide.

The growing challenge of bacterial resistance to antibiotics has led to the development of several options of conservative wound treatment. None of them have shown the combined wound healing attributes found in medical grade honey. Studies [16] have showed that a range of species of bacteria, including MRSA and vancomycin resistant enterococci, are susceptible to the antibacterial activity of honey without developing resistance. Although the type of honey and concentration of honey dressing varied throughout the studies, the conclusion of successful inhibition of antibacterial growth remains [1].

The use of Manuka honey requires a minimum of medical training, has negligible adverse effects, is relatively inexpensive and in remote locations an ideal first-aid dressing to prevent infections before other treatment including surgery is obtained [10].

Although many encouraging clinical observations and small studies have been done, further research is needed [17]. Considering the adverse effects of conventional antibiotics and the increasing resistance to antibiotics, we hope that Manuka honey soon finds a more permanent place in the physician’s arsenal of wound treatment products.

## 4 Conclusion

We successfully treated a penile denudation with Manuka honey after an unsuccessful split-thickness skin graft. Surgical wounds, ulcers, and burns may be infected and can be challenging, time consuming, and expensive to treat. Manuka honey may be a good alternative to reconstructive surgical procedures and can be managed on an out-patient basis.

## Sources of funding

All authors declare that we did not receive any funding for our research.

## Ethical approval

This is not considered a research study. This is a descriptive case report.

## Consent

We confirm that written and signed consent has been obtained prior to submission from the patient.

## Author contribution

**Amalie Sylvester-Hvid:** Drafting, revision, approval of final manuscript.

**Magnus Balslev Avnstorp:** Data collection, revision, approval of final manuscript.

**Tobias Fjeld:** Drafting, revision, approval of final manuscript.

**Bjørn Crewe:** Data collection, revision, approval of final manuscript.

## Registration of research studies

This is not considered a human study. This is a descriptive case report.

## Guarantor

Amalie Sylvester-Hvid, MD.

Magnus Balslev Avnstorp, MD.

## Provenance and peer review

Not commissioned, externally peer-reviewed.

## Declaration of Competing Interest

All authors declare no conflict of interests financially or personally.
